# Cardamonin Attenuates Inflammation and Oxidative Stress in Interleukin-1β-Stimulated Osteoarthritis Chondrocyte through the Nrf2 Pathway

**DOI:** 10.3390/antiox10060862

**Published:** 2021-05-27

**Authors:** Yi-Jen Peng, Jeng-Wei Lu, Chian-Her Lee, Herng-Sheng Lee, You-Hsiang Chu, Yi-Jung Ho, Feng-Cheng Liu, Chun-Jung Huang, Chia-Chun Wu, Chih-Chien Wang

**Affiliations:** 1 Department of Pathology, National Defense Medical Center, Tri-Service General Hospital, Taipei 114, Taiwan; yijen0426@gmail.com (Y.-J.P.); olive_leaf@hotmail.com.tw (C.-J.H.); 2 Department of Biological Sciences, National University of Singapore, Singapore 117543, Singapore; dbslujw@nus.edu.sg; 3 Department of Orthopedics, School of Medicine, College of Medicine, Taipei Medical University Hospital, Taipei Medical University, Taipei 110, Taiwan; lee060008@tmu.edu.tw; 4 Laboratory Medicine, Department of Pathology, Kaohsiung Veterans General Hospital, Kaohsiung 813, Taiwan; herngsheng131419@gmail.com; 5 National Defense Medical Center, Graduate Institute of Life Sciences, Taipei 114, Taiwan; joseph12896@tmu.edu.tw (Y.-H.C.); ejho@mail.ndmctsgh.edu.tw (Y.-J.H.); 6 National Defense Medical Center, School of Pharmacy, Taipei 114, Taiwan; 7 Department of Medicine, Rheumatology/Immunology and Allergy, National Defense Medical Center, Tri-Service General Hospital, Taipei 114, Taiwan; lfc10399@mail.ndmctsgh.edu.tw; 8 Department of Orthopedics, National Defense Medical Center, Tri-Service General Hospital, Taipei 114, Taiwan; doc20281@mail.ndmctsgh.edu.tw

**Keywords:** osteoarthritis, cardamonin, oxidative stress, chondrocyte, interleukin-1 beta

## Abstract

Osteoarthritis (OA) is a chronic degenerative joint disease characterized by the deterioration of articular cartilage. The progression of OA leads to an increase in inflammatory mediators in the joints, thereby promoting the destruction of the cartilage matrix. Recent studies have reported on the anti-inflammatory and antioxidant properties of cardamonin, which also appears to interact with cellular targets, such as nuclear erythroid 2-related factor 2 (Nrf2), extracellular signal-regulated kinase (ERK), and mammalian target of rapamycin (mTOR) during the progression of tumors. To date, few studies have investigated the effects of cardamonin on chondrocyte inflammation. In the current study, we determined that treating interleukin-1 beta (IL-1β-stimulated chondrocyte cells) with cardamonin significantly reduced the release of nitric oxide (NO) and prostaglandin E2 (PGE2) and significantly inhibited the expression of pro-inflammatory proteins, including inducible nitric oxide synthase (iNOS) and cyclooxygenase 2 (COX2). Cardamonin was also shown to: (1) inhibit the activation and production of matrix metalloproteinases (MMPs), (2) suppress the nuclear factor-κB (NF-κB) signaling pathway, (3) suppress the expression of toll-like receptor proteins, (4) activate the Nrf2 signaling pathway, and (5) increase the levels of antioxidant proteins heme oxygenase-1 (HO-1) and NAD(P)H:quinone oxidoreductase 1 (NQO1). The increase in antioxidant proteins led to corresponding antioxidant effects (which were abolished by Nrf2 siRNA). Our findings identify cardamonin as a candidate Nrf2 activator for the treatment and prevention of OA related to inflammation and oxidative stress.

## 1. Introduction

Osteoarthritis (OA) is a degenerative joint disease, a leading cause of chronic disability, and an important national health issue [1,2]. In roughly half of afflicted individuals, OA restricts normal daily activities and greatly reduces quality of life. OA can affect the entire joint structure, including articular cartilage, subchondral bone, synovium, tendons, muscle, and meniscus fibrocartilage tissue in the knee joints [3,4]. The disease has been attributed to an imbalance between the synthesis and degradation of the extracellular matrix (ECM) [5,6]. Furthermore, increased inflammatory mediators in joints during OA progression contributes to the destruction of the cartilage matrix [4]. Nonetheless, recent cartilage repair treatments have the potential to not only relieve pain and improve quality of life but also to delay or eliminate the need for joint replacement [7]. Most approaches to OA management focus on pain relief using systemic or local drugs, physical therapies, or surgery [8]. However, current therapeutic options have a number of major drawbacks, such as applicability to limit size defect, long rehabilitation times, and a lack of readily available graft material [9,10].

Inflammatory cytokines and catabolic factors hinder the functions of chondrocytes and promote the development of OA [3]. One inflammatory cytokine, interleukin-1 beta (IL-1β) has been identified in osteoarthritic synovial fluid and can trigger a succession of cartilage related catabolic effects [11]. This process directly activates nuclear factor kappa-B (NF-κB) and mitogen-activated protein (MAP) kinase, which up-regulates the expression of cartilage matrix-degrading enzymes, such as cyclooxygenase 2 (COX2), inducible nitric oxide synthase (iNOS), and matrix metalloproteinases (MMPs) [12]. These cytokines are elevated in joint disorders and are closely related to pathological conditions that lead to the production of nitric oxide (NO) and prostaglandin E_2_ (PGE_2_). Previous research identified NO and PGE_2_ (both of which are highly expressed in the synovial fluid of OA patients) as therapeutic targets in the treatment of OA [13,14]. However, there are many adverse effects associated with non-steroidal anti-inflammatory drugs (NSAIDs) [15,16]. Thus, inhibiting IL-1β-stimulated inflammatory mediators via natural sources is the preferred approach to OA treatment.

In recent years, natural plant extracts have been demonstrated to have anti-inflammatory and antioxidant effects. Cardamonin (2′,4′-dihydroxy-6′-methoxychalcone) is a chalcone isolated primarily from Zingiberaceae which has been reported to exert anti-inflammatory and antioxidant properties [17]. Cardamonin interacts with various cellular signaling targets, such as nuclear erythroid 2-related factor 2 (Nrf2), extracellular signal-regulated kinase (ERK), and mammalian target of rapamycin (mTOR), during the progression of tumors [18]. These interactions provide evidence that the mechanism behind the anti-tumor activities of cardamonin involves negatively modulating the signal transducer and activator of transcription (STAT) family [19]. Cardamonin has also recently been reported to attenuate the expression of COX2 and iNOS by reducing p65/nuclear factor-κB (NF-κB) nuclear translocation and inhibiting phosphorylation of nuclear factor kappa B (IκB) [20]. Our objectives in the current study were to investigate the protective effects of cardamonin on human chondrocytes in OA models and to identify the mechanism which underlies these protective effects.

## 2. Materials and Methods

### 2.1. Primary Culture

Human articular cartilage tissues were obtained from OA patients who underwent total knee replacement. Residual osteoarthritic cartilage was first removed from the joint surfaces, and articular cartilage was then cut into small fragments and incubated with antimicrobial solution (500 IU/mL penicillin/streptomycin) (Gibco, Carlsbad, CA, USA) for 3 h. Subsequently, articular cartilage was washed with phosphate buffered saline (PBS) (Gibco, Carlsbad, CA, USA) and digested. Articular chondrocyte extraction was then performed via sequential enzymatic digestion at 37 °C under 5% CO_2_ with 0.25% trypsin (Gibco, Carlsbad, CA, USA) for 30 min followed by 3 mg/mL blend collagenase type H (Sigma-Aldrich, Merck KGaA, Darmstadt, Germany) for 12 h. The samples were checked for digestion by examination under a BX43 light microscope (Olympus Corp, Tokyo, Japan), and the cell suspension was collected and filtered through nylon mesh using a sterile pastette. After centrifugation at 1000 rpm for 10 min, the supernatant was discarded, and the pellet was resuspended in 10 mL Dulbecco’s Modified Eagle’s medium (DMEM)/Nutrient Mixture F-12 HAM medium (Gibco, Carlsbad, CA, USA) supplemented with 10% fetal bovine serum (FBS) (Gibco, Carlsbad, CA, USA), 100 international units (IU)/mL penicillin, and 100 µg/mL streptomycin. Finally, the pellet was cultured under a humidified 5% CO_2_ atmosphere at 37 °C. Cells between passages 2 and 3 were used in subsequent experiments.

### 2.2. Cell Viability

Chondrocyte viability was determined using the 3-(4,5-dimethylthiazol-2-yl)-2,5-diphenyltetrazolium bromide (MTT) assay (Roche, Indianapolis, IN, USA). For this, primary human OA chondrocytes were seeded in 200 µL complete medium in a 96-well plate at a density of 10,000 cells per well. At confluence, chondrocytes were serum starved overnight and then treated with various concentrations of cardamonin (1, 5, 10, 20, 40 µg/mL) (Catalog number: C8249; Sigma-Aldrich, St. Louis, MO, USA) in serum free medium for 24 or 48 h. Chondrocytes were subsequently treated with 20 µl of MTT at 0.5 mg/mL (Sigma-Aldrich, St. Louis, MO, USA) for 3 h and formazan crystal solubilized using dimethyl sulfoxide (DMSO) at 150 µL (Sigma-Aldrich, St. Louis, MO, USA). Absorbance was recorded at 570 nm using a Synergy HT plate reader (Bio-Tek Instruments Inc., Winooski, VT, USA).

### 2.3. Reactive Oxygen Species (ROS) Measurement

ROS was measured via 2′,7′-dichlorofluorescein-diacetate (H2DCF-DA) (Sigma-Aldrich, St. Louis, MO, USA) staining in accordance with the manufacturer’s instructions and then observed using a microscope. For the measurement of IL-1β induced ROS, chondrocytes were pretreated with SBE fractions (50 µg/mL) for 2 h, labeled with H2DCF-DA (20 µM) for 0.5 h, and then stimulated with IL-1β for 5 min. Detailed protocol for ROS detection has been previously described by Khan et al. [21].

### 2.4. Griess Reaction

NO concentrations in synovial fluid can be derived from their stable end product, nitrite, measured using the Griess reaction. Briefly, we incubated an aliquot of joint fluid or cultured medium with 50 µL of 1% sulphanilamide (Sigma-Aldrich, St. Louis, MO, USA) in 5% phosphoric acid (Sigma-Aldrich, St. Louis, MO, USA) and 50 µL of 0.1% N-1-naphthylethylenediamine dihydrochloride (Sigma-Aldrich, St. Louis, MO, USA). After 20 min incubation at room temperature, absorbance was using a microplate reader to measure at a wavelength of 550 nm (BioTek Instruments, Winooski, VT, USA).

### 2.5. Extraction of Protein and Western Blotting Analysis

Both of the cells and tissues were immediately washed using PBS and lysed in situ for 15 min with radioimmunoprecipitation assay (RIPA) lysis buffer (Thermo Fisher Pierce, Waltham, MA, USA) containing 100 µM Na_3_VO_4_, and 100× protease inhibitor cocktail (Thermo Fisher Pierce, Waltham, MA, USA). Following centrifugation for 15 min at 13,000 rpm, whole cell lysates were collected, and the protein concentration was determined using the Lowry method. Equal amounts of protein were then loaded onto 10% sodium dodecyl sulfate (SDS)-polyacrylamide gel and transferred to polyvinylidene fluoride (PVDF) membranes (Merck KGaA, Darmstadt, Germany). PVDF membranes were incubated with bovine serum albumin (BSA) at 2%. (Sigma-Aldrich, St. Louis, MO, USA) in TBST (12.5 mM Tris/HCl, pH 7.6, 137 mM NaCl, 0.1% Tween 20) (Sigma-Aldrich, St. Louis, MO, USA) at 4 °C overnight. After washing with TBST three times, blots were incubated with primary antibodies diluted in TBST. After washing with TBST three more times, the blots were incubated with horseradish peroxidase (HRP) labeled secondary antibodies at room temperature for 1 h. Membranes were then rewashed thoroughly, and the binding results were detected using the enhanced chemiluminescence plus Western blotting detection system (Thermo Fisher Pierce, Waltham, MA, USA) in accordance with the manufacturer’s instructions. Finally, membranes were scanned and subjected to densitometry analysis (VisionWorks LS, UVP, CA, USA) in accordance with the manufacturer’s protocols. Specific antibodies that we used are listed in Supplementary Table S1.

### 2.6. Enzyme-Linked Immunosorbent Assay (ELISA)

To detect cytokine expression levels in tissue fluid and cultured medium in accordance with the manufacturer’s protocols we used an ELISA kit (R&D Systems, Minneapolis, MN, USA).

### 2.7. Gelatin Zymography

The extract was mixed with sample buffer solution containing SDS, glycerol, and bromophenol blue (Sigma-Aldrich, St. Louis, MO, USA, respectively). Equal quantities of each sample were separated on SDS-polyacrylamide gel (8%) containing 1 mg/mL gelatin (Sigma-Aldrich, St. Louis, MO, USA). After performing SDS-polyacrylamide gel electrophoresis, the gels were washed twice using 2.5% Triton X-100 (Sigma-Aldrich, St. Louis, MO, USA) for 30 min to remove SDS and then twice more with distilled water. The gels were subsequently equilibrated with incubation buffer (100 mM Tris/HCl, 30 mM CaCl_2_, 0.01% NaN3) (Sigma-Aldrich, St. Louis, MO, USA) and then incubated with incubation buffer for 20 h at 37 °C prior to staining with Coomassie Blue solution for 40 min. Destaining was performed in methanol/acetic acid/distilled water.

### 2.8. Safranin O-Fast Green Staining

Slides were preheated for 30 min at 75 °C, then dewaxed and hydrated in an ethanol gradient of 70%, 80%, 95%, or 100% (Sigma-Aldrich, St. Louis, MO, USA). Following this, slides were stained with Weigert’s iron hematoxylin working solution (Sigma-Aldrich, St. Louis, MO, USA) for 5 min at room temperature and washed gently under running tap water for 5 min before being stained with 0.02% Fast Green solution (Sigma-Aldrich, St. Louis, MO, USA) for 5 min. Slides were then rinsed in 1% acetic acid (Sigma-Aldrich, St. Louis, MO, USA) for 10 s then transferred to 1% safranin O solution (Sigma-Aldrich, St. Louis, MO, USA) for 3 min. Finally, slides were carefully rinsed twice in fresh 95% ethanol and air dried.

### 2.9. Electrophoretic Mobility Shift Assay (EMSA)

The DNA binding activity of NF-κB, AP-1, and STAT-3, EMSA was evaluated using a LightShift Chemiluminescence RNA EMSA Kit (Thermo Fisher Scientific, Waltham, MA, USA). For this, biotin end-label duplex DNA was incubated with nuclear extract and electrophoresed on native gel. The reaction mixtures were separated using 5% native polyacrylamide gel at 4 °C for 1 h before being transferred to a nylon membrane (GE Healthcare, Buckinghamshire, UK). Biotin end-labeled DNA was tested with a streptavidin–HRP conjugate and enhanced chemiluminescence (ECL) reagents. Finally, the membranes were exposed to UV light for 10 min and analyzed using the UVP AutoChemi Image System (UVP Inc., Upland, CA, USA).

### 2.10. SiRNA Transfection

We obtained siNrf2 and control siRNA from Applied Biosystems (Thermo Fisher Pierce, Waltham, MA, USA). Primary chondrocyte cells were seeded in 6-well plates and transfected with siNrf2 or control siRNA using lipofectamine 2000 reagent (Invitrogen, Waltham, MA, USA) in accordance with the manufacturer’s instructions. The cells were then cultured for 24 h prior to use in the experiments.

### 2.11. Measurement of ROS and Immunofluorescence Assay

Quantification of the inhibitory effect of cardamonin upon cellular oxidative stress was incubated with H2DCF-DA for 30 min which is a cell-permeable probe used to detect intracellular ROS. Following incubation, cells were gently washed twice with PBS to remove unbound H2DCF-DA. Cells were then cultured with IL-1β in chamber slides at a density of 1 × 10^4^ cells for 5 min. To measure the intracellular expression of ROS, we observed emissions under excitation at a wavelength of 485 nm using an BX61 immunofluorescence microscope (Olympus Corp, Tokyo, Japan).

### 2.12. Statistical Analysis

All statistical analysis was conducted using Prism 8.0 (GraphPad Software Inc., San Diego, CA, USA) and Image J software (NIH, Bethesda, MD, USA). All data were obtained from at least three independent experiments, and values were expressed as mean ± standard deviation (SD). Statistical evaluation of quantification data pertaining to mRNA and protein was performed using Student’s *t*-tests. The results were considered significant at a *p*-value less than 0.05.

## 3. Results

### 3.1. Effects of Cardamonin on Cell Viability and NO_2_^−^ Production

**Figure 1:**
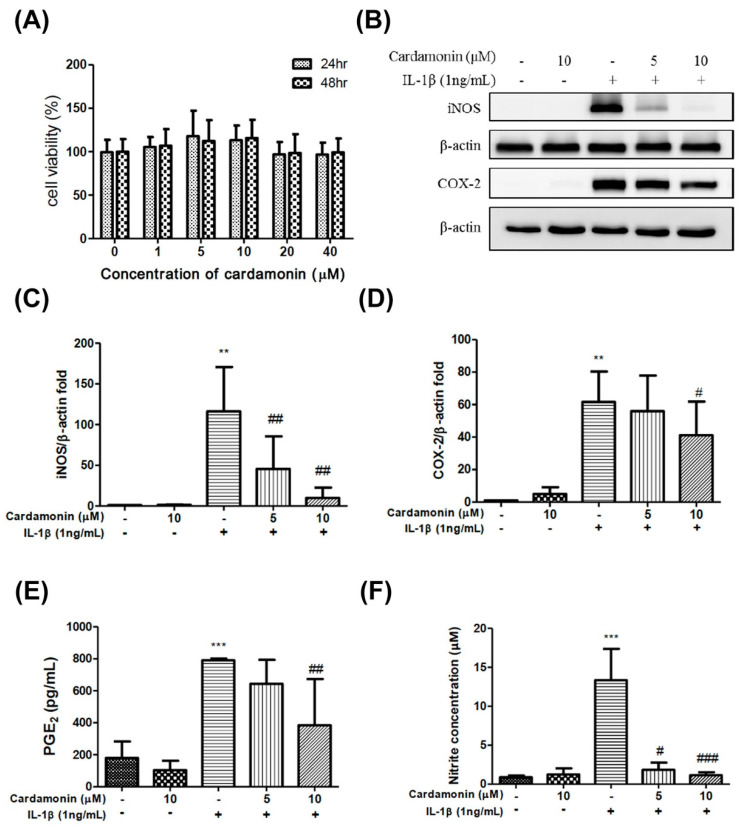
Cardamonin suppressed IL-1β-induced iNOS and COX2 expression and prevented the IL-1β-mediated induction of PGE2 and the IL-1β-mediated production of nitric oxide in human OA chondrocytes. (**A**) MTT assays were used to examine the cytotoxicity of cardamonin on human chondrocytes incubated with 0, 1, 5, 10, 20, and 40 µM/mL of cardamonin for 24 or 48 h. (**B**–**D**) At a concentration of 10 µM, cardamonin was shown to significantly reduce iNOS and COX-2 protein expression levels in chondrocytes stimulated with IL-1β for 24 h. (**E**,**F**) PGE2 and nitric oxide concentrations were significantly increased following treatment with IL-1β for 24 h; however, these increases were significantly decreased following treatment with 10 µM cardamonin. All data comes from at least three independent experiments, and their significance were as follows: cardamonin group and the control group are indicated by an asterisk (*); cardamonin group and the IL-1β group are indicated by a hash symbol (#). # *p* < 0.05; **, ## *p* < 0.01; *** *p* < 0.001.

Evaluation of the cytotoxicity of cardamonin on human chondrocytes by MTT assays. For this, chondrocytes were incubated with cardamonin at 0, 1, 5, 10, 20 or 40 µM concentrations. Seed in a 96-well plate at a density of 1 × 10^4^ cells/well for the primary chondrocytes. Assays revealed that the presence of cardamonin did not significantly affect cell viability (Figure 1A).

Elevated iNOS and COX-2 protein expression levels were observed in chondrocytes incubated with IL-1 for 24 h. (Figure 1B). Nonetheless, concentrations of 5 or 10 µM cardamonin were showed to suppress iNOS expression (whereby expression levels were comparable to those observed in cells that had not undergone IL-1β stimulation) (Figure 1C). However, cardamonin only suppressed COX2 expression at a concentration of 10 µM (Figure 1D).

PGE_2_ is a pro-inflammatory mediator that is produced under stimulation with IL-1β, and iNOS is generated NO from the amino acid L-arginine. In the current study, NO status was assessed by measuring iNOS, nitrites, and nitrate levels. The inhibitory effects of cardamonin on NO release was correlated with the effects on iNOS expression, as determined by the Griess reaction. At concentrations of 5 or 10 µM, cardamonin was shown to have a significant inhibitory effect on PGE_2_ (Figure 1E) and NO (Figure 1F). Taken together, these results indicate that the inhibitory capacity of cardamonin against NO production in IL-1β-stimulated chondrocyte cells is likely due to the inhibition of iNOS protein expression.

### 3.2. Cardamonin Affected IL-1β-Stimulated MMP Activity in Chondrocytes

**Figure 2:**
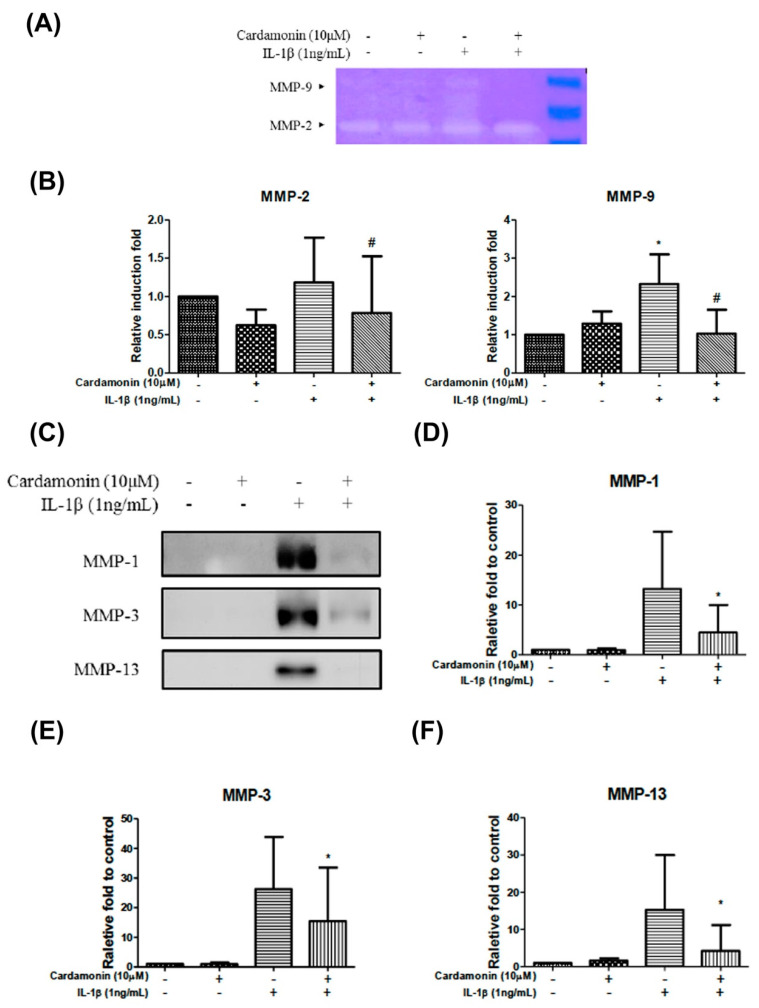
Cardamonin suppressed the activities of MMP-2 and MMP-9 as well as the protein expression levels of MMP-1, MMP-3 and MMP-13 in human OA chondrocytes induced by IL-1β. (**A**,**B**) Gel images revealed the analysis of cardamonin effects on MMP-2 and MMP-9 activities of IL-1β insulted using gelatin zymography gel. (**C**–**F**) Protein expression levels of MMP-1, MMP-3 and MMP-13 in IL-1β -induced chondrocytes following treatment with 10 µM cardamonin for 24 h. MMP-1, MMP-3 and MMP-13 protein expression demonstrate the analysis of cardamonin effects with IL-1β treatment, and significantly reduced with cardamonin. All data comes from at least three independent experiments, and their significance were as follows: cardamonin group and the control group are indicated by an asterisk (*); cardamonin group and the IL-1β group are indicated by a hash symbol (#). *, # *p* < 0.05.

We also sought to determine whether cardamonin suppresses the expression of MMP-1, MMP-2, MMP-3, MMP-9, and MMP-13. Gelatin zymography gels revealed that treatment with cardamonin at a concentration of 10 µM for 24 h inhibited the increase in MMP-2 and MMP-9 expression induced by IL-1β (Figure 2A,B). Western blot analysis was further used to examine MMP-1, MMP-3, and MMP-13 protein expression levels (Figure 2C,D). Results showed that treatment with cardamonin at a concentration of 10 µM for 24 h mediated IL-1β-induced increases in MMP-1 (Figure 2D), MMP-3 (Figure 2E), and MMP-13 (Figure 2F).

### 3.3. Cardamonin Suppressed the Loss of Extra Cellular Matrix and ROS Generation Induced by IL-1β Stimulation

**Figure 3:**
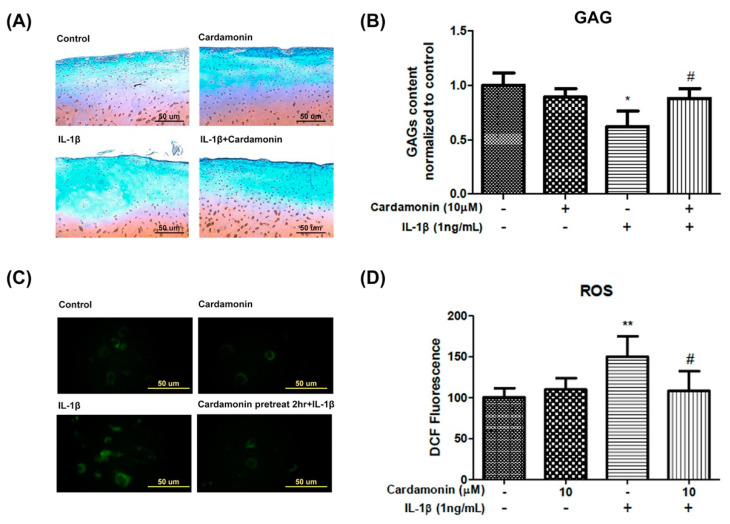
Effects of cardamonin on IL-1β-induced cartilage defects and ROS generation in human OA chondrocytes. (**A**,**B**) Cardamonin significantly decreased IL-1β-induced cartilage defects in chondrocytes. (**C**,**D**) Cardamonin suppressed IL-1β-stimulated generation of ROS in chondrocytes. Following pretreatment with cardamonin for 2 h, cells were stimulated using IL-1β. A fluorescence microscope and Image J software were used to assess ROS fluorescence intensity and perform quantitative analysis. All data comes from at least three independent experiments, and their significance were as follows: cardamonin group and the control group are indicated by an asterisk (*); cardamonin group and the IL-1β group are indicated by a hash symbol (#). *, # *p* < 0.05; ** *p* < 0.01.

Safranin O-fast green staining and ROS fluorescence were respectively used to examine the effects of cardamonin on the extra cellular matrix and ROS levels. IL-1β treatment was shown to significantly reduce glycosaminoglycan (GAG) content (Figure 3A). However, pretreatment with cardamonin at a concentration of 10 µM inhibited this effect (Figure 3B). In addition, cells were incubated with 20 µM H2DCF-DA for 30 min and then subjected to IL-1β insult for 5 min to induce ROS expression. ELISA results revealed that IL-1β treatment significantly increased ROS expression (Figure 3C), but pretreatment with cardamonin at a concentration of 10 µM for 2 h significantly inhibited this effect (Figure 3D).

### 3.4. Cardamonin Inhibited IL-1β-Stimulated NF-κB, AP-1, STAT-3 and Nrf2 Activities in Chondrocytes

**Figure 4:**
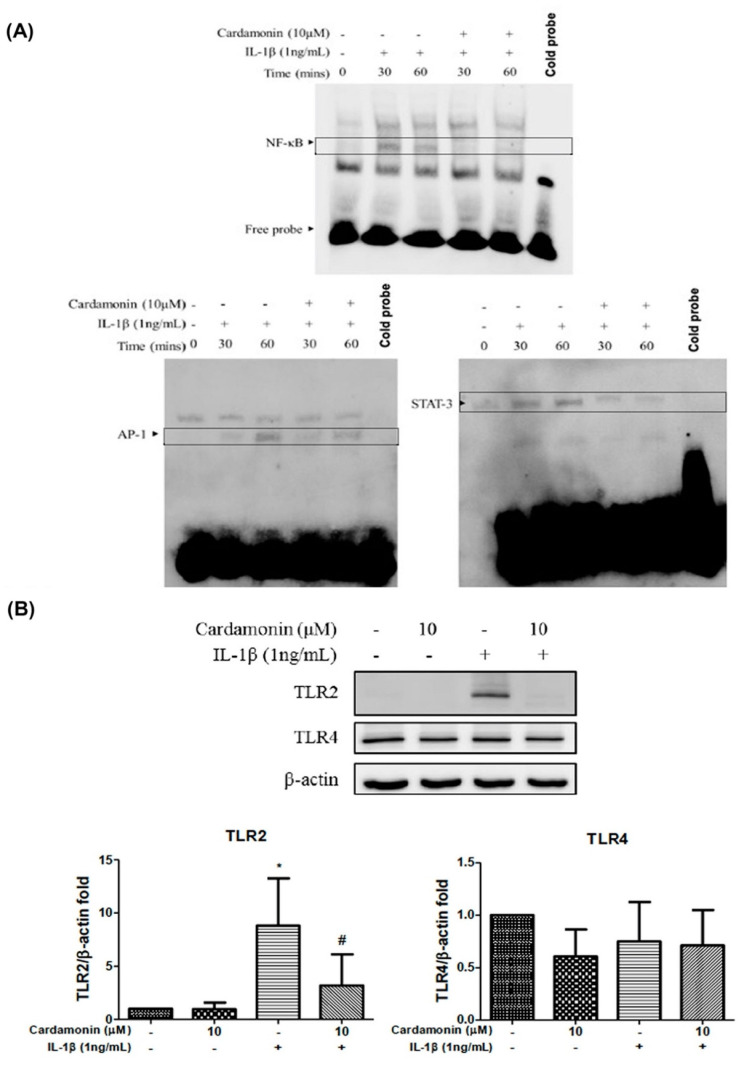

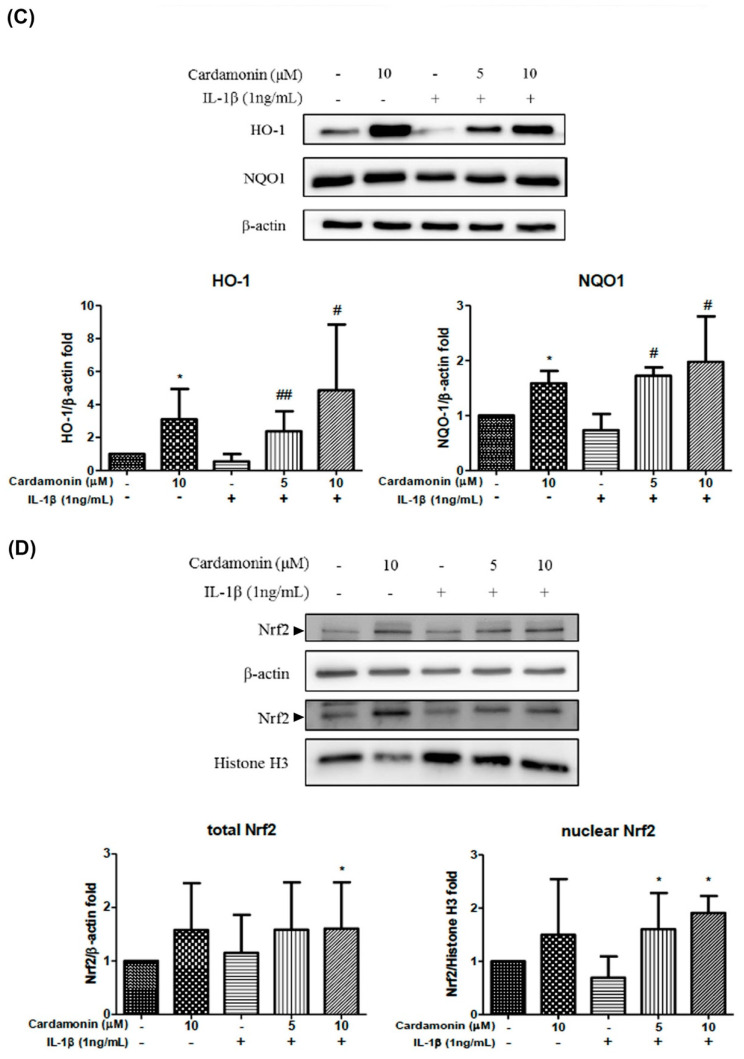
Effects of cardamonin on the activation of nuclear factor. (**A**) Protein samples from human OA chondrocyte cultures were detected using EMSA assays. (**B**) Chondrocytes were incubated with cardamonin at a concentration of 10 µM for 24 h and then either stimulated with IL-1β or not to detect protein expression levels of TLR2 and TLR 4 receptors. (**C**,**D**) Primary chondrocyte cells were incubated with cardamonin at a concentration of 10 µM for 24 h and then either stimulated with IL-1β or not to detect protein expression levels of HO-1, NQO1, total Nrf2 and nuclear Nrf2. Specifically, we sought to examine the effects of cardamonin on protein expression levels of HO-1, NQO1, total Nrf2, and nuclear Nrf2 following IL-1β treatment. All data comes from at least three independent experiments, and their significance were as follows: cardamonin group and the control group are indicated by an asterisk (*); significant differences between the cardamonin group and the IL-1β group are indicated by a hash symbol (#). *, # *p* < 0.05; ## *p* < 0.01.

We also examined the inhibitory effects of cardamonin on NF-κB-binding at the level of transcriptional activity. Treatment with IL-1β (1 ng/mL) for 30 or 60 min stimulated NF-κB transcription beyond baseline levels. Pretreating chondrocytes with 10 µM cardamonin inhibited the IL-1β-induced transcription of NF-κB, AP-1, and STAT-3 (Figure 4A). We also observed an increase in multiple alarmins, degradation products of cartilage extracellular matrix proteins, proteoglycans constituents, free fatty acids, and other danger-associated molecular patterns (DAMPs) which induced multiple inflammatory cytokines, mediated in large by signaling through various pattern recognition receptors (PRRs) expressed in OA cartilage and synovium, including toll-like receptor 2 (TLR2) and toll-like receptor 4 (TLR4). Pretreatment with cardamonin at a concentration of 10 µM was shown to significantly inhibit the IL-1β -stimulated production of TLR2 (Figure 4B).

Oxidative stress plays an important role in inflammation; HO-1 is an antioxidative protein which is expressed during amelioration of inflammation. To examine the effect of cardamonin on HO-1 and NQO1 expression under IL-1β stimulation we used Western blot analysis (Figure 4C). At present, the mechanism by which cardamonin mediates ROS expression is unclear; however, it is known that Nrf2 is an important regulator of ROS production in cardamonin-inhibited inflammation. Treatment with cardamonin at a concentration of 5 or 10 µM significantly up-regulated the expression levels of both total and nuclear Nrf2 proteins (Figure 4D).

### 3.5. Nrf2 Inhibition Counteracted the Effects of Cardamonin

**Figure 5:**
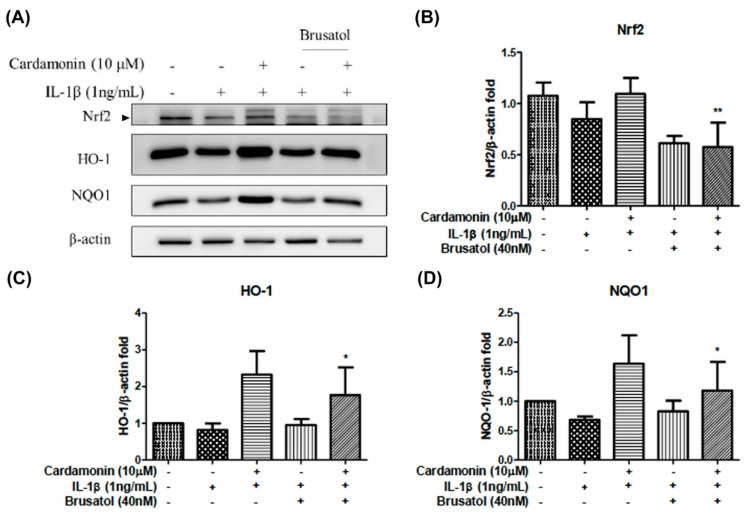
Effects of cardamonin on the HO-1, and NQO1 proteins expression and activation of the Nrf2 pathway on human OA chondrocytes stimulated using brusatol. (**A**) Representative Western blotting gel illustrating the effects of cardamonin-treated cells stimulated with IL-1β and brusatol. (**B**–**D**) Nrf2, HO-1, and NQO1 illustrating the effects of cardamonin effects on protein expression levels in chondrocytes stimulated using IL-1β and brusatol. All data comes from at least three independent experiments, and their significance were as follows: cardamonin and baseline group and the IL-1β are indicated by an asterisk (*). * *p* < 0.05; ** *p* < 0.01.

We further elucidated the role of the Nrf2 signaling pathway in the inhibition effects of cardamonin. Note that the HO-1 and NQO1 enzymes are located downstream from Nrf2, that these enzymes are mediators of Nrf2, and that brusatol is an inhibitor of the Nrf2 pathway. In this study, to examine the effects of brusatol on the activation of Nrf2 and downstream proteins of HO-1 and NQO1 we also used Western blot analysis (Figure 5A). Co-treatment with 40 nM brusatol and 10 µM cardamonin significantly elevated the protein expression of Nrf2 (Figure 5B), HO-1 (Figure 5C), and NQO1 (Figure 5D) in IL-1β-stimulated chondrocytes.

### 3.6. Nrf2 siRNA Abolished the Effects of Cardamonin

**Figure 6:**
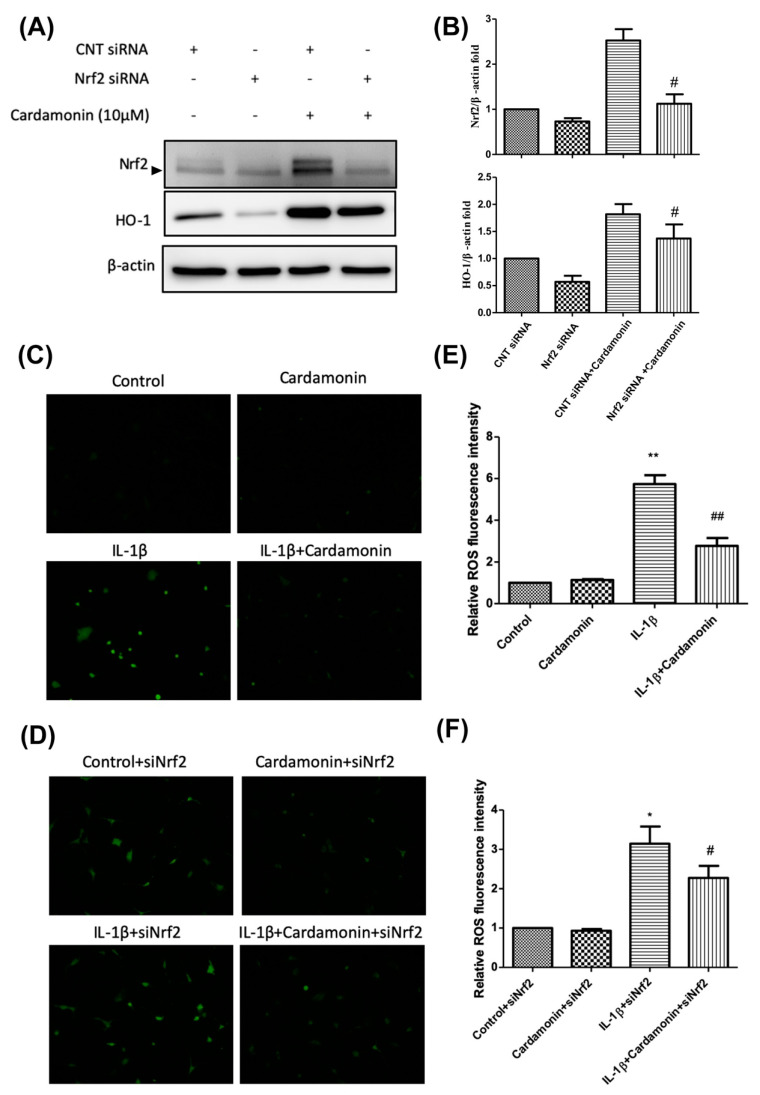
Inhibited HO-1 expression and induced ROS generation in IL-1β-stimulated human OA chondrocytes were used Nrf2 siRNA. (**A**) Representative Western blot analysis illustrating the effects of incubating chondrocytes with CNT-siRNA, Nrf2-siRNA, CNT-siRNA+ cardamonin, or Nrf2-siRNA+ cardamonin for 2 h. (**B**) Nrf2 and HO-1 protein expression levels showing the effects of cardamonin treatment in chondrocytes. (**C**,**D**) Chondrocytes were incubated with cardamonin, siNrf2 for 2 h, and then stimulated with IL-1β for 2 h. Treatment with cardamonin alone or cardamonin with siNrf2 knock down suppressed IL-1β-stimulated ROS generation in chondrocytes. (**E**,**F**) ROS generation was detected and quantified in terms of fluorescence intensity. All data comes from at least three independent experiments, and their significance were as follows: IL-1β group and the groups treated with cardamonin alone or with cardamonin + siNrf2 knock down are indicated by an asterisk (*) or a hash symbol (#). *, # *p* < 0.05; **, ## *p* < 0.01.

We established an Nrf2 gene knockdown model via Nrf2 siRNA transfection in chondrocytes. We then used Western blot analysis to examine the effects of cardamonin on Nrf2 and HO-1 protein expression (Figure 6A). Our results revealed that Nrf2 siRNA knockdown suppressed Nrf2 protein expression as well as the increase in Nrf2 and HO-1 protein levels induced by pretreatment with cardamonin (Figure 6B). Further identification of the level of ROS was detected. Cardamonin significantly down-regulated the level of ROS expression in IL-1β-stimulated chondrocyte cells (Figure 6C,E). Moreover, an Nrf2 siRNA knockdown effect was also detected. It was not affected the stimulated with IL-1β-stimulated ROS expression. However, the expression of ROS showed significant attenuation in Nrf2 siRNA knockdown, combined with 10 µM concentration of cardamonin pretreatment and IL-1β-stimulation (Figure 6D,F). These results indicate that the expression of antioxidant proteins Nrf2 and HO-1 play important roles in the anti-inflammatory effects of cardamonin.

### 3.7. Cardamonin-Suppressed Dephosphorylation of the Cell Signaling Pathway by IL-1β

**Figure 7:**
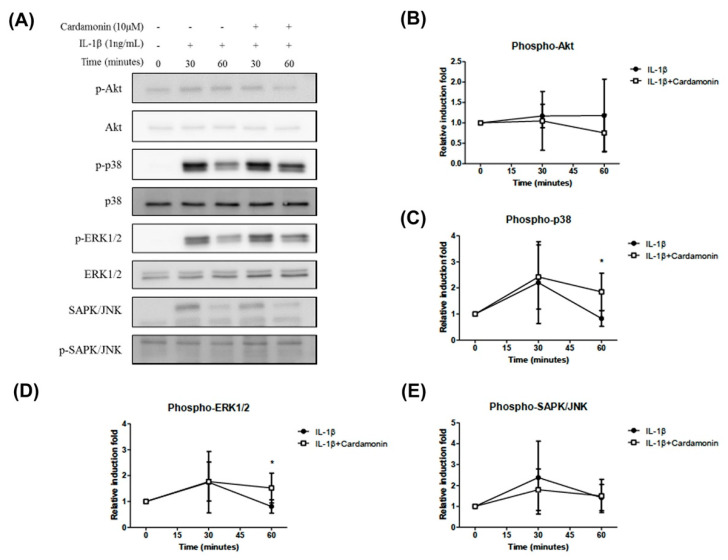
Effects of cardamonin on the activation of the p38/ERK signaling pathway in IL-1β-induced human OA chondrocytes. (**A**) Representative Western blot analysis illustrating the effects of pretreating chondrocytes with cardamonin at a concentration of 10 µM for 0, 30 and 60 min prior to stimulation with IL-1β. (Effects of treatment with 10 µM cardamonin on untreated cells are also shown.) The phosphorylation of Akt, p38, ERK1/2, and SAPK/JN was detected in chondrocytes. (**B**–**E**) Akt, p38, ERK1/2, and SAPK/JN showed the effect of cardamonin on the phosphorylation level of IL-1β treatment, while the effect of cardamom on p38 and ERK1/2 was significantly reduced. All data comes from at least three independent experiments, and their significance were as follows: cardamonin group and the IL-1β group are indicated by an asterisk (*). * *p* < 0.05.

Activation of the mitogen-activated protein kinase (MAPK) and phosphatidylinositol 3-kinase (PI3K)/protein kinase B (AKT) signaling pathway may lead to the phosphorylation of the Nrf2 protein and its translocation into the nucleus. Western blot analysis revealed that stimulation with IL-1β for 30 min led to the phosphorylation of p38, extracellular signal-regulated protein kinase (ERK1/2), and stress-activated protein kinase/c-Jun NH(2)-terminal kinase (SAPK/JNK) (Figure 7A). Cardamonin treatment was not shown to inhibit the activation of AKT following 30 min of stimulation with IL-1β, but did delay its activation in 60 min (Figure 7B). Pretreatment with cardamonin was also shown to delay IL-1β induced dephosphorylation of p38 and ERK1/2 (Figure 7C,D). However, pretreatment with cardamonin did not affect SAPK/JNK phosphorylation (Figure 7E). Taken together, these results suggest that cardamonin inhibited IL-1β-stimulated NO production while also providing positive feedback promoting the production of antioxidants and anti-inflammatory agents.

## 4. Discussion

OA is a complex joint pathology that leads to chronic disability. The precise mechanism of OA pathogenesis has not been elucidated, and no effective treatment has been developed to block the progression of the disease. Increasing evidence suggests that cardamonin possesses anti-inflammatory and antioxidant properties. This study reports two major findings. Firstly, cardamonin inhibited the overexpression of iNOS (Figure 1B,C), COX2 (Figure 1B,D), and MMPs (Figure 2) in chondrocytes stimulated by IL-1β. Secondly, cardamonin reduced the generation of ROS in chondrocytes (Figure 3C,D) by activating the Nrf2 pathway (Figure 4, Figure 5 and Figure 6) via p38 (Figure 6A,C) and ERK (Figure 6A,D). Previous studies have reported that the mediators secreted in the early stages of OA are proinflammatory cytokines, including IL-1β and tumor necrosis factor-alpha (TNF-α). This evidence shows that IL-1β plays a central role in the pathogenesis of OA; therefore, it is commonly used to induce OA in vitro in chondrocytes. IL-1β stimulation induces the expression of COX2, which in turn increases the synthesis of PGE2. PGE2 has been implicated in bone resorption and joint pain in cases of OA [22]. PGE2 and NO are both capable of upregulating the production of MMPs and other inflammatory cytokines, and inhibiting the IL-1β-stimulated production of inflammatory mediators has proven useful in the treatment of OA. In this study, pretreatment with cardamonin significantly decreased IL-1β-stimulated PGE2 (Figure 1E) and NO (Figure 1F) production by attenuating the expression of iNOS (Figure 1B,C) and COX2 (Figure 1B,D). These observations indicate that the expression of iNOS and COX2 could be regulated by the anti-inflammatory effects of cardamonin in articular chondrocytes.

OA is characterized by the destruction of articular cartilage due to an imbalance between the biosynthesis and degradation of the extra cellular matrix (ECM). Previous studies have demonstrated that MMPs play a crucial role in the degradation of articular cartilage. MMPs are proteolytic enzymes commonly expressed in joint disorders. They have been shown to degrade the ECM and are therefore viewed as a promising pharmacological target for the treatment of OA. The fact that MMP-1, MMP-3, and MMP-13 are primarily found in cartilage indicates their probable participation in OA progression. We therefore sought to determine whether cardamonin could be used to inhibit the expression and/or activity of MMPs and inflammatory mediators induced in chondrocytes by IL-1β. MMP-2 and MMP-9 activity was investigated using gelatin zymography in order to examine the effects of cardamonin treatment following IL-1β stimulation (Figure 2A). In previous studies, IL-1β was shown to induce the expression of MMP-1, MMP-3, MMP-13, and ADAMTS-4 in human tendon cells [23]. In the current study, we detected the IL-1β induced expression of MMP-1, MMP-2, MMP-3, MMP-9, and MMP-13 in articular chondrocytes (Figure 2). For MMP-9 activities detection, IL-1β showed a significant increase after 24 h treatment. However, when cells were pretreated with cardamonin prior to IL-1β stimulation, the production of MMP-1 (Figure 2D), MMP-3 (Figure 2E), and MMP-13 (Figure 2F) was significantly decreased compared with cells that were only stimulated with IL-1β and did not receive cardamonin pretreatment. Cardamonin was also shown to suppress the activities of MMP-2 and MMP-9 (Figure 2A,B). This suggests that cardamonin (1) exerts protective effects on chondrocytes and (2) has a potential role in treating cartilage damage in cases of OA.

ROS are small reactive molecules derived from molecular oxygen. They have been linked to oxidative stress and inflammatory response in OA [24]. ROS degrades a variety of GAGs, such as chondroitin sulfate (CS), hyaluronic acid (HA), and dermatan sulfate [25]. Potential sources of ROS identified in chondrocytes, such as NO synthase, can modulate chondrocyte behavior and extracellular matrix homeostasis in OA patients. In the current study, we investigated the effects of cardamonin on the activation of iNOS and ROS in IL-1β-stimulated chondrocytes with the aim of elucidating the mechanism which underlies the antioxidation effects of cardamonin. In previous studies, IL-1β-stimulated chondrocytes were shown to induce high iNOS and ROS levels. In the current study, pretreatment with cardamonin attenuated IL-1β-stimulated iNOS activation (Figure 2B,C) and ROS expression (Figure 3C,D) in chondrocytes.

Nrf2 is a member of the cap ‘n’ collar (CNC) transcription factor subfamily [26]. Previous studies which employed Nrf2-knockout mice reported increased mRNA and protein levels for COX2, iNOS, interleukin-6 (IL-6), and TNF-α. Nonetheless, the activation of Nrf2 could lead to nuclear translocation, which should decrease the level of COX2 and iNOS expression. Research has shown that Nrf2-dependent antioxidant genes HO-1 and NQO-1 can block TNF-α and IL-6 inflammatory mediators. Previous studies have also reported that HO-1 expression can be enhanced by various oxidative-inducing lipopolysaccharides (LPS) [27]. We found that cardamonin inhibited the expression of inflammatory proteins (COX2 and iNOS) and inflammatory cytokines (PGE2) while increasing the expression of antioxidant proteins HO-1 and NQO1. We also found that cardamonin treatment dramatically inhibited the increase in HO-1 levels induced by IL-1β (Figure 4C). For IL-1β stimulation, overproduction or overexpression of HO-1 (Figure 5A,C) was reduced with the IL-1β-stimulated ROS in the articular chondrocyte culture medium (Figure 6A,B). Taken together, these results indicate that the anti-inflammatory effects of cardamonin can be attributed to activation of the Nrf2 pathway.

High iNOS, COX2, and MMP expression levels in arthritic joints is due to the activation of a tightly regulated and synchronized signaling cascade which is activated by IL-1β and involves the mitogen-activated protein kinases (MAPKs) signaling pathway [28,29]. Using cardamonin to suppress NO generation via iNOS phosphorylation delayed the activation of p38 and ERK1/2 kinases. Recent studies have shown that NO activates ERK signaling through the down-regulation of MAP kinase phosphatase. MAPKs are activated through the phosphorylation of specific tyrosine and threonine residues by upstream kinases in response to inflammatory signals. In the current study, we demonstrated that IL-1β enhances the translocation of NF-ΚB and the activation of the p38 and ERK signaling pathways (Figure 7C,D). Interestingly, pretreating chondrocyte cells with cardamonin suppressed the IL-1β-induced release of MMPs, iNOS, and PGE2, as indicated by the attenuation of their corresponding gene expressions. This was at least partly achieved through the blocking of NF-κB and p38 activation. These findings suggest that cardamonin could be a novel Nrf2 activator and a useful nutritional supplement in OA therapy.

## 5. Conclusions

The findings in this study revealed that cardamonin decreased the expression of iNOS and the release of NO, which in turn reduced oxidative stress. Cardamonin was also shown to play an important role as an Nrf2 activator with effects against IL-1β induced inflammation. These results suggest that cardamonin could be used as a potential agent candidate against OA.

## Data Availability

Data is contained within the article or supplementary material.

## Supplementary Materials

The following are available online at https://www.mdpi.com/article/10.3390/antiox10060862/s1, Table S1: The antibodies were used in Western blotting for the present study.
