# Supplementary material for: Cardamonin Attenuates Inflammation and Oxidative Stress in Interleukin-1β-Stimulated Osteoarthritis Chondrocyte through the Nrf2 Pathway
Source: Antioxidants (Basel). 2021 May 27;10(6):862. doi: 10.3390/antiox10060862 (PMC8227809; doi:10.3390/antiox10060862)
Supplement: Supplementary file 1 [file antioxidants-10-00862-s001.zip › antioxidants-1225874-supplementary.pdf]

antioxidants

Supplementary Material

Table S1. The antibodies were used in western blotting for the present study.

Name	Catalog Number	Company
Anti-MMP-13	ab53015	Abcam, Cambridge, UK
Anti-TLR2	ab191458	Abcam, Cambridge, UK
Anti-TLR4	ab13867	Abcam, Cambridge, UK
Anti-HO-1	ab52947	Abcam, Cambridge, UK
Anti-NQO1	62262	Cell Signaling Technology, Danvers, MA, USA
Anti-Nrf2	#12721	Cell Signaling Technology, Danvers, MA, USA
Anti-Akt	#9272	Cell Signaling Technology, Danvers, MA, USA
Anti-p-Akt	#9271	Cell Signaling Technology, Danvers, MA, USA
Anti-p38	#9212	Cell Signaling Technology, Danvers, MA, USA
Anti-p-p38	#9211	Cell Signaling Technology, Danvers, MA, USA
Anti-ERK1/2	#9102	Cell Signaling Technology, Danvers, MA, USA
Anti-p-ERK1/2	#9101	Cell Signaling Technology, Danvers, MA, USA
Anti-SAPK/JNK	#9252	Cell Signaling Technology, Danvers, MA, USA
Anti-p-SAPK/JNK	#9251	Cell Signaling Technology, Danvers, MA, USA
Anti-Histone H3	4499	Cell Signaling Technology, Danvers, MA, USA
Anti-βactin	sc-47778	Santa Cruz Biotechnology, Dallas, TX, USA
Anti-rabbit IgG HRP	P0217	DAKO, CA, USA
Anti-mouse IgG (HRP)	P0161	DAKO, CA, USA
